# Effect of aesthetic nursing interventions on the health-related quality of life of cervical cancer patients after treatment

**DOI:** 10.3389/fsurg.2026.1609977

**Published:** 2026-05-29

**Authors:** Haizhen Tan, Xi Yang

**Affiliations:** Department of Gynecology, The Central Hospital of Enshi Prefecture Tujia and Miao Autonomous Prefecture, Enshi City, Hubei Province, China

**Keywords:** aesthetic nursing, anxiety, body image, cervical cancer, quality of life

## Abstract

**Objective:**

To evaluate the effects of aesthetic nursing interventions on postoperative quality of life, body image, and psychological outcomes in patients with cervical cancer.

**Methods:**

This retrospective cohort study included 210 patients undergoing cervical cancer surgery between January 2022 and June 2024. Patients were categorized into an aesthetic nursing intervention group (*n* = 90) and a basic nursing group (*n* = 120). Outcomes were assessed using the EORTC QLQ-C30 as the primary quality-of-life measure, with the SF-36 as a supplementary generic health-status measure, together with the BIS, BIDQ, GAD-7, and PHQ-9 at 2 weeks, 1 month, 3 months, and 6 months postoperatively. Between-group differences were analyzed using non-parametric tests, and longitudinal changes were evaluated using linear mixed-effects models. Exploratory multivariable regression was conducted to assess time-dependent effects.

**Results:**

Patients receiving aesthetic nursing demonstrated significantly better quality of life scores (EORTC QLQ-C30) and improved body image and anxiety outcomes compared with the basic nursing group (*P* < 0.05). Improvements were more pronounced during the mid- to late postoperative period. Regression analysis indicated that aesthetic nursing was associated with improved outcomes over time, particularly after the early postoperative phase.

**Conclusions:**

Aesthetic nursing interventions may improve postoperative quality of life, body image, and anxiety in patients with cervical cancer. Further prospective studies are needed to confirm these findings.

## 1 Introduction

Cervical cancer is one of the most common malignant tumors among women worldwide, and its incidence rate and mortality are still at a high level in some countries and regions (1, 2). With the popularization of screening and vaccination, the early diagnosis and treatment of cervical cancer have significantly improved, resulting in an increasing survival rate for patients year by year (3–5). However, the treatment process, especially surgical treatment, can have a profound impact on the patient's physical health and psychological state (6, 7). After surgery, patients not only need to face the recovery of physical function, but may also experience significant psychological pressure, such as changes in body image, decreased self-identity, and negative emotions such as anxiety and depression, which greatly affect the patient's health-related quality of life and long-term recovery (8, 9).

Aesthetic nursing intervention is a comprehensive nursing model that focuses on the physiological, psychological, and social needs of patients, aiming to enhance their body image identification and self-efficacy, and help them better cope with the challenges during postoperative recovery (10, 11). This nursing model emphasizes not only appearance-related care but also the psychological and emotional needs of patients. Cervical cancer and its treatment have been shown to affect multiple domains of health-related quality of life, including pain, physical functioning, emotional well-being, and psychosocial identity. Surgical treatment may result in persistent pain, functional limitations, and visible bodily changes, which in turn negatively influence body image and psychosocial adjustment. In addition to physical symptoms, changes in appearance and perceived femininity may also lead to emotional distress and reduced self-esteem, particularly among postoperative patients. Compared with routine nursing care, aesthetic nursing interventions place greater emphasis on appearance management, psychological support, and the reconstruction of self-image. Therefore, from a theoretical perspective, such interventions may have beneficial effects on body image, psychosocial adjustment, and postoperative quality of life (12).

Because postoperative recovery in cervical cancer involves multiple domains, including global quality of life, body image, and psychological symptoms, more than one validated instrument was used to characterize these outcomes. The EORTC QLQ-C30 served as the primary instrument for assessing global health status/quality of life. The SF-36 was included only as a supplementary generic measure of overall health status, whereas the BIS, BIDQ, GAD-7, and PHQ-9 were used to provide more specific evaluation of body image and psychological symptoms.

This study aimed to evaluate whether aesthetic nursing intervention was associated with postoperative quality of life, body image, and psychological outcomes in patients with cervical cancer compared with routine nursing care.

## 2 Materials and methods

### 2.1 Patients

This study adopted a retrospective cohort design, collecting cervical cancer patient data from hospital medical records and follow-up databases between January 2022 and June 2024. Our hospital treats an average of approximately 180 patients with cervical cancer per year, of whom about 60% are eligible for surgical treatment. This design offers high clinical relevance and feasibility, aligning closely with the objectives of this study. The inclusion criteria were as follows: (1) age ≥18 years; (2) pathologically confirmed cervical cancer; and (3) completion of surgical treatment with available postoperative follow-up data. The exclusion criteria were as follows: (1) severe psychiatric disorders or cognitive impairment that could interfere with questionnaire completion; (2) other active malignancies; (3) pregnancy or lactation; and (4) missing key baseline or follow-up outcome data. All cases were screened according to the above inclusion and exclusion criteria, and a total of 210 cases were finally included, with 120 patients in the basic nursing group and 90 patients in the aesthetic nursing group. The specific case screening process is shown in Supplementary Figure S1.

### 2.2 Intervention methods

#### 2.2.1 Routine care

Patients in the basic nursing group received routine postoperative care, including pain management, infection prevention, and guidance on functional exercise. Routine nursing was delivered according to standardized procedures by experienced nursing staff. Nursing guidance and evaluation were provided once daily after surgery, with each session lasting approximately 30 min. After discharge, follow-up was conducted weekly by telephone or face-to-face visit for 6 months.

#### 2.2.2 Aesthetic nursing

Patients in the aesthetic nursing group received aesthetic nursing in addition to basic nursing care, with major components including scar camouflage, skin management, appearance-related guidance, and psychological support. Specific components involved guiding patients in the use of medical skincare products such as scar patches and silicone gel; teaching the proper application of concealing cosmetics, such as concealer and foundation, to scarred areas; and providing basic skincare routines to reduce postoperative pigmentation and dryness. Clothing and styling advice focused on addressing sensitivity around abdominal incisions by recommending comfortable, loose-fitting garments with good coverage. Patients were also instructed on how to use clothing color, cut, and layering to divert visual attention, thereby enhancing their overall appearance and confidence. Based on changes in body shape, patients were guided on how to improve body proportions through clothing choices. In addition, appropriate makeup tips were offered—such as eyebrow shaping and techniques to enhance eye expression—along with hairstyle suggestions or guidance on wearing wigs to address appearance-related changes. Psychological counseling and mindfulness training were delivered twice weekly during hospitalization, with each session lasting approximately 30 min. After discharge, sessions were held once per week during the first month (30–45 min per session), and then biweekly thereafter (30–45 min per session). The intervention focused on common issues such as postoperative anxiety, body image distress, and changes in family roles. Supportive dialogue aimed to improve emotional expression and coping ability, while patients were guided in mindfulness exercises such as breathing, mindful eating, and body scanning to help them stay present and relieve anxiety. All intervention providers held nationally accredited psychological counseling certifications or had passed hospital-organized professional training and assessments. To ensure consistency and reproducibility, all staff received unified training before implementation, used a standardized operating manual, and underwent regular supervision and evaluation throughout the intervention process. The intervention protocol was implemented in all patients in the aesthetic nursing group during hospitalization, and follow-up intervention was delivered according to the planned schedule whenever feasible during the postoperative follow-up period.

### 2.3 Data collection

Baseline patient information collected before the nursing intervention included basic demographic characteristics, such as age, education level, marital status, and economic status. Disease-related and surgical information were also recorded, including cancer stage, type of surgery, surgical approach, intraoperative complications, and duration of surgery. Recovery status and psychological outcomes were assessed at 2 weeks, 1 month, 3 months, and 6 months after surgery. The assessment instruments included the EORTC QLQ-C30, SF-36, Body Image Scale (BIS), Body Image Disturbance Questionnaire (BIDQ), GAD-7, and PHQ-9 (13–15). Educational level was categorized as low (primary school or below), medium (secondary school or technical school), or high (college degree or above). Economic status was categorized as <5,000 RMB, 5,000–8,000 RMB, or >8,000 RMB.

All data were collected by two researchers who had received unified training and were not directly involved in routine clinical nursing care. Prior to data collection, the researchers underwent standardized training that covered interview procedures, questionnaire administration protocols, the use of standardized instructions, and the handling of common participant questions. Baseline demographic and clinical data were obtained through medical record review and patient interviews conducted by the researchers, with each interview lasting approximately 10–15 min. Postoperative assessments were performed at 2 weeks, 1 month, 3 months, and 6 months after surgery, either in inpatient wards or during scheduled follow-up clinic visits. Data were primarily collected through patient self-completed questionnaires, with guidance provided by the researchers when necessary. To ensure data quality, standardized assessment procedures and instructions were applied at all time points, and completed questionnaires were reviewed on site for completeness and accuracy.

For all instruments, scores were calculated according to their respective published scoring manuals. The EORTC QLQ-C30 global health status/quality of life scale was transformed to a 0–100 scale, with higher scores indicating better quality of life. The SF-36 total score was used as a supplementary measure of overall health status, with higher scores indicating better health status. For the BIS, BIDQ, GAD-7, and PHQ-9, higher scores indicated worse body image disturbance or more severe psychological symptoms. The primary outcome was global health status/quality of life assessed using the EORTC QLQ-C30. Secondary outcomes included body image and psychological well-being, evaluated using the Body Image Scale (BIS), Body Image Disturbance Questionnaire (BIDQ), and the Generalized Anxiety Disorder-7 (GAD-7) and Patient Health Questionnaire-9 (PHQ-9). The SF-36 was used as a supplementary generic measure of overall health status.

### 2.4 Statistical analysis

Data were analyzed using R version 4.4.1. Continuous variables were expressed as median (interquartile range, IQR), and categorical variables as frequency (percentage). Between-group comparisons were performed using the Mann–Whitney *U*-test for continuous variables and the chi-square test or Fisher's exact test for categorical variables, as appropriate.

To evaluate longitudinal changes over time, linear mixed-effects models were applied, with nursing method, time, and their interaction included as fixed effects, and patient ID as a random effect to account for repeated measurements. Time was treated as a categorical variable.

In addition, an exploratory multivariable linear regression analysis was conducted to assess time-dependent changes before and after the observed 2-week postoperative turning point. The models included nursing method, time segments (pre- and post-2 weeks), their interaction terms, and baseline imbalanced variables (surgical approach and intraoperative complications) as covariates.

Results are presented as regression coefficients (β), standard errors, and *P* values. A two-sided *P* < 0.05 was considered statistically significant.

## 3 Results

### 3.1 Differences in baseline information between the basic nursing intervention group and the aesthetic nursing intervention group

As shown in Table 1, there was no statistically significant difference (*P* > 0.05) between the two groups of patients in terms of age, educational level, marital status, economic status, cancer stage, type of surgery, duration of surgery, and health-related indicators (EORTC QLQ-C30, SF-36, BIS, BIDQ, GAD-7, and PHQ-9). However, there were significant differences in surgical approach (*P* = 0.011) and intraoperative complications (*P* = 0.022). The aesthetic nursing group had a lower proportion of laparoscopic surgery and fewer intraoperative complications than the basic nursing group. These findings suggest that the two groups were generally comparable at baseline, although differences were observed in surgical approach and intraoperative complications. These baseline imbalances were therefore adjusted for in the regression analyses and further explored in the stratified analyses.

**Table 1:** Baseline information of postoperative patients with cervical cancer.

Variables	All patients (*n* = 210)	Basic nursing intervention (*n* = 120)	Aesthetic nursing intervention (*n* = 90)	*P*-Value
Age	41.53 (25.08–54.89)	41.53 (25.08–54.63)	41.55 (25.85–54.89)	0.787
Education level				
Low	10 (4.76%)	7 (5.83%)	3 (3.33%)	0.085
Medium	71 (33.81%)	47 (39.17%)	24 (26.67%)	
High	129 (61.43%)	66 (55%)	63 (70%)	
Marital status				
Married	168 (80%)	101 (84.17%)	67 (74.44%)	0.160
Single	26 (12.38%)	13 (10.83%)	13 (14.44%)	
Separated/Partnered but not married	16 (7.62%)	6 (5%)	10 (11.11%)	
Economic status				
<5,000 RMB	114 (54.29%)	72 (60%)	42 (46.67%)	0.054
5,000–8,000 RMB	74 (35.24%)	40 (33.33%)	34 (37.78%)	
>8,000 RMB	22 (10.48%)	8 (6.67%)	14 (15.56%)	
Cancer stage				
Stage I	132 (62.86%)	70 (58.33%)	62 (68.89%)	0.155
Stage II	78 (37.14%)	50 (41.67%)	28 (31.11%)	
Surgical type				
Radical surgery	193 (91.9%)	106 (88.33%)	87 (96.67%)	0.053
Conservative surgery	17 (8.1%)	14 (11.67%)	3 (3.33%)	
Surgical approach				
Laparoscopic surgery	138 (65.71%)	88 (73.33%)	50 (55.56%)	0.011
Open surgery	72 (34.29%)	32 (26.67%)	40 (44.44%)	
Intraoperative complications				
Yes	16 (7.62%)	14 (11.67%)	2 (2.22%)	0.022
No	194 (92.38%)	106 (88.33%)	88 (97.78%)	
Duration of surgery (min)	179 (106–257)	175 (106–257)	183 (108–252)	0.583
EORTC QLQ-C30	45 (39–52)	45 (39–52)	45 (40–51)	0.342
SF-36	45 (40–51)	46 (40–51)	45 (40–50)	0.892
Body Image Scale	20 (15–26)	21 (15–26)	20 (15–25)	0.438
Body Image Disturbance Questionnaire	27 (23–32)	27 (23–32)	26 (23–30)	0.471
GAD-7	8 (6–11)	8 (6–10)	9 (6–11)	0.914
PHQ-9	4 (3–6)	4 (3–6)	4 (3–6)	0.468

### 3.2 Changes in postoperative health-related quality of life, body image cognition, and mental health of cervical cancer patients

Overall, the aesthetic nursing intervention group showed significant advantages across multiple indicators at different postoperative time points (2 weeks, 1 month, 3 months, and 6 months). In terms of health-related quality of life (EORTC QLQ-C30 and SF-36), scores in the aesthetic nursing group were consistently higher than those in the basic nursing group at all postoperative time points, with statistically significant differences (*P* < 0.05) and effect sizes all exceeding 0.3. BIS and BIDQ scores showed greater improvement in the aesthetic nursing group during postoperative recovery, with effect sizes reaching moderate or higher levels. The largest effect size for the Body Image Disturbance Questionnaire was observed at 3 months post-surgery [Cohen's d = 0.573, 95% CI (0.293, 0.851)]. For psychological outcomes, the aesthetic nursing group showed more favorable anxiety outcomes, with the largest effect size observed for GAD-7 at 2 weeks post-surgery [Cohen's d = 0.714, 95% CI (0.432, 0.995)]. Differences in PHQ-9 scores were more modest and were observed mainly at later follow-up time points (Table 2), whereas the adjusted regression model suggested that depressive symptom changes were primarily time-related rather than intervention-specific.

**Table 2:** Changes in patients’ mental health and health-related quality of life over time following aesthetic nursing interventions.

Outcome	All patients (*n* = 210)	Basic nursing intervention (*n* = 120)	Aesthetic nursing intervention (*n* = 90)	*P*-value	Cohen's d	CI-Low	CI-High
EORTC QLQ-C30							
Before the intervention	45 (39–52)	45 (39–52)	45 (40–51)	0.342	0.108	−0.165	0.382
2 weeks post-surgery	39 (36–43)	39 (36–43)	40 (36–43)	0.0214	−0.334	−0.609	−0.058
1 month post-surgery	45 (42–47)	44 (42–47)	45 (42–47)	0.001	−0.481	−0.758	−0.204
3 months post-surgery	54 (48–60)	53 (48–59)	55 (48–60)	0.015	−0.335	−0.610	−0.060
6 months post-surgery	67 (62–75)	67 (62–73)	69 (62–75)	0.013	−0.349	−0.624	−0.073
SF-36							
Before the intervention	45 (40–51)	46 (40–51)	45 (40–50)	0.892	0.197	−0.077	0.471
2 weeks post-surgery	41 (38–45)	40 (38–44)	42 (38–45)	<0.001	−0.635	−0.914	−0.354
1 month post-surgery	50 (46–54)	49 (46–54)	51 (46–54)	0.003	−0.439	−0.715	−0.162
3 months post-surgery	60 (54–65)	58 (54–64)	61 (54–65)	<0.001	−0.556	−0.834	−0.277
6 months post-surgery	69 (66–74)	68 (66–72)	71 (66–74)	<0.001	−0.485	−0.762	−0.207
Body image scale							
Before the intervention	20 (15–26)	21 (15–26)	20 (15–25)	0.438	0.227	−0.047	0.501
2 weeks post-surgery	22 (18–26)	22 (18–26)	21 (18–26)	0.092	0.228	−0.046	0.502
1 month post-surgery	14 (12–17)	15 (12–17)	14 (12–17)	0.009	0.364	0.088	0.639
3 months post-surgery	11 (8–14)	12 (8–14)	10 (8–14)	0.003	0.421	0.144	0.697
6 months post-surgery	4 (2–7)	5 (2–7)	4 (2–7)	0.002	0.435	0.158	0.711
Body image disturbance questionnaire							
Before the intervention	27 (23–32)	27 (23–32)	26 (23–30)	0.471	0.129	−0.145	0.402
2 weeks post-surgery	31 (28–35)	32 (28–35)	31 (28–35)	<0.001	0.529	0.250	0.806
1 month post-surgery	22 (18–25)	23 (18–25)	21 (18–25)	0.012	0.384	0.107	0.659
3 months post-surgery	13 (10–17)	14 (10–17)	12 (10–17)	<0.001	0.573	0.293	0.851
6 months post-surgery	10 (8–13)	11 (8–13)	10 (8–13)	0.010	0.376	0.100	0.651
GAD-7							
Before the intervention	8 (6–11)	8 (6–10)	9 (6–11)	0.914	−0.253	−0.527	0.022
2 weeks post-surgery	13 (11–15)	13 (11–15)	12 (11–15)	<0.001	0.714	0.432	0.995
1 month post-surgery	9 (7–11)	10 (7–11)	8 (7–11)	0.002	0.448	0.171	0.724
3 months post-surgery	6 (4–8)	7 (4–8)	6 (4–8)	0.007	0.394	0.118	0.670
6 months post-surgery	2 (0–5)	3 (0–5)	2 (0–5)	<0.001	0.476	0.198	0.753
PHQ-9							
Before the intervention	4 (3–6)	4 (3–6)	4 (3–6)	0.468	−0.076	−0.350	0.197
2 weeks post-surgery	8 (6–10)	9 (6–10)	8 (6–10)	0.092	0.263	−0.012	0.537
1 month post-surgery	5 (3–6)	5 (3–6)	4 (3–6)	0.220	0.165	−0.109	0.439
3 months post-surgery	2 (0–4)	2 (0–4)	2 (0–4)	0.033	0.279	0.004	0.554
6 months post-surgery	2 (0–3)	2 (0–3)	1 (0–3)	0.044	0.271	−0.004	0.545

### 3.3 Exploratory multivariable linear regression analysis across postoperative periods

As shown in Table 2, the trajectories of postoperative outcomes suggested that the least favorable values for quality of life, body image, and psychological measures occurred at approximately 2 weeks after surgery. Based on this observed pattern, an exploratory multivariable linear regression analysis was performed to examine changes before and after the 2-week postoperative turning point. Nursing method, time segment, and their interaction terms were entered into the models, while surgical approach and intraoperative complications were included as adjustment variables because of their baseline imbalance.

For quality of life outcomes, both the EORTC QLQ-C30 and SF-36 showed a similar temporal pattern, with poorer scores during the early postoperative phase and progressive recovery thereafter. In the adjusted models, aesthetic nursing was associated with better overall EORTC QLQ-C30 scores (*β* = 2.546, *P* = 0.006) and SF-36 scores (*β* = 2.547, *P* = 0.014). Before the turning point, score trajectories indicated deterioration in both EORTC QLQ-C30 (*β* = −1.124, *P* < 0.001) and SF-36 (*β* = −3.566, *P* = 0.023), whereas after the turning point both measures improved over time (EORTC QLQ-C30: *β* = 2.723, *P* < 0.001; SF-36: *β* = 2.451, *P* < 0.001). Moreover, significant post-turning-point interactions were observed for both instruments (EORTC QLQ-C30: *β* = 2.374, *P* = 0.039; SF-36: *β* = 2.259, *P* < 0.001), suggesting greater improvement during later recovery in the aesthetic nursing group.

Body image outcomes showed a related but not identical pattern. For the BIS, worse body image burden was observed during the early postoperative period, followed by improvement over time. Aesthetic nursing was associated with lower BIS scores overall (*β* = −1.848, *P* = 0.031), and the post-turning-point interaction remained significant (*β* = −1.017, *P* < 0.001), indicating greater improvement after the early postoperative phase. For the BIDQ, aesthetic nursing was likewise associated with lower disturbance scores overall (*β* = −1.056, *P* = 0.011). In contrast to the BIS pattern, the significant interaction was observed before the turning point (*β* = −2.802, *P* < 0.001), suggesting that the intervention-related advantage in body-image disturbance emerged earlier for BIDQ than for BIS. Across models, body-image scores worsened before the turning point (BIS: *β* = 1.986, *P* < 0.001; BIDQ: *β* = 1.920, *P* < 0.001) and improved thereafter (BIS: *β* = −2.708, *P* < 0.001; BIDQ: *β* = −3.319, *P* < 0.001).

Among psychological outcomes, the intervention effect appeared clearer for anxiety than for depressive symptoms. For GAD-7, aesthetic nursing was associated with lower anxiety scores overall (*β* = −0.939, *P* = 0.008), and a significant interaction was identified before the turning point (*β* = −3.981, *P* = 0.001), indicating a greater reduction in early postoperative anxiety in the aesthetic nursing group. Although anxiety scores were higher during the early postoperative phase (*β* = 4.243, *P* < 0.001), they declined significantly thereafter (*β* = −1.678, *P* < 0.001). By contrast, for PHQ-9, no significant association was found between aesthetic nursing and depressive symptom scores (*P* = 0.398), and no significant interaction with time was observed. Instead, PHQ-9 changes were mainly characterized by a time effect, with depressive symptoms increasing before the turning point (*β* = 1.039, *P* = 0.014) and decreasing thereafter (*β* = −0.994, *P* < 0.001). Surgical approach and intraoperative complications were not significantly associated with most modeled outcomes. (Table 3).

**Table 3:** Exploratory multivariable linear regression analysis of postoperative outcomes associated with aesthetic nursing intervention.

Outcome	Variables	Estimate	Std error	Statistic	*P* value
EORTC QLQ-C30	Time Pre-Break	−1.124	0.321	−3.471	<0.001
	Method	2.546	0.918	2.775	0.006
	Time Post Break	2.723	0.066	41.428	<0.001
	Surgical Approach	0.255	0.206	1.238	0.216
	Intraoperative Complications	−0.065	0.368	−0.176	0.860
	Time Pre-Break × Method	0.117	0.100	1.166	0.244
	Method × Time Post Break	2.374	1.147	2.070	0.039
SF-36	Time Pre-Break	−3.566	1.568	−2.274	0.023
	Method	2.547	1.040	2.448	0.014
	Time Post Break	2.451	0.074	33.330	<0.001
	Surgical Approach	0.259	0.234	1.109	0.268
	Intraoperative Complications	−0.018	0.418	−0.042	0.967
	Time Pre-Break × Method	0.006	0.113	0.057	0.954
	Method × Time Post Break	2.259	0.431	5.242	<0.001
Body Image Scale	Time Pre-Break	1.986	0.078	25.429	<0.001
	Method	−1.848	0.860	−2.146	0.031
	Time Post Break	−2.708	0.061	−44.122	<0.001
	Surgical Approach	0.031	0.193	0.158	0.874
	Intraoperative Complications	0.097	0.345	0.282	0.778
	Time Pre-Break × Method	0.617	1.980	0.312	0.755
	Method × Time Post Break	−1.017	0.094	−10.824	<0.001
Body Image Disturbance Questionnaire	Time Pre-Break	1.920	0.193	9.948	<0.001
	Method	−1.056	0.416	−2.538	0.011
	Time Post Break	−3.319	0.087	−38.233	<0.001
	Surgical Approach	0.223	0.273	0.814	0.416
	Intraoperative Complications	−0.240	0.488	−0.491	0.624
	Time Pre-Break × Method	−2.802	0.801	−3.498	<0.001
	Method × Time Post Break	0.072	0.133	0.544	0.587
GAD-7	Time Pre-Break	4.243	0.265	15.986	<0.001
	Method	−0.939	0.354	−2.652	0.008
	Time Post Break	−1.678	0.036	−46.610	<0.001
	Surgical Approach	0.022	0.114	0.193	0.847
	Intraoperative Complications	−0.101	0.204	−0.497	0.619
	Time Pre-Break × Method	−3.981	1.170	−3.403	0.001
	Method × Time Post Break	−0.003	0.055	−0.061	0.951
PHQ-9	Time Pre-Break	1.039	0.423	2.461	0.014
	Method	0.401	0.474	0.845	0.398
	Time Post Break	−0.994	0.034	−29.392	<0.001
	Surgical Approach	−0.001	0.107	−0.012	0.991
	Intraoperative Complications	0.058	0.190	0.306	0.760
	Time Pre-Break × Method	−1.313	1.091	−1.203	0.229
	Method × Time Post Break	0.005	0.052	0.096	0.924

### 3.4 Linear mixed-effects model analysis of longitudinal changes in quality of life between the two groups

For the EORTC QLQ-C30, significant group-by-time interactions were observed at 3 and 6 months postoperatively, indicating greater improvement in global health status/quality of life during the mid- to late postoperative period in the aesthetic nursing group. For the supplementary SF-36 analysis, significant group-by-time interactions were observed at all postoperative follow-up time points. Overall, these findings were consistent with the main regression results and support the potential benefit of aesthetic nursing during postoperative recovery (Supplementary Table S1).

### 3.5 Exploratory analysis of overall change patterns during follow-up

**Figure 1:**
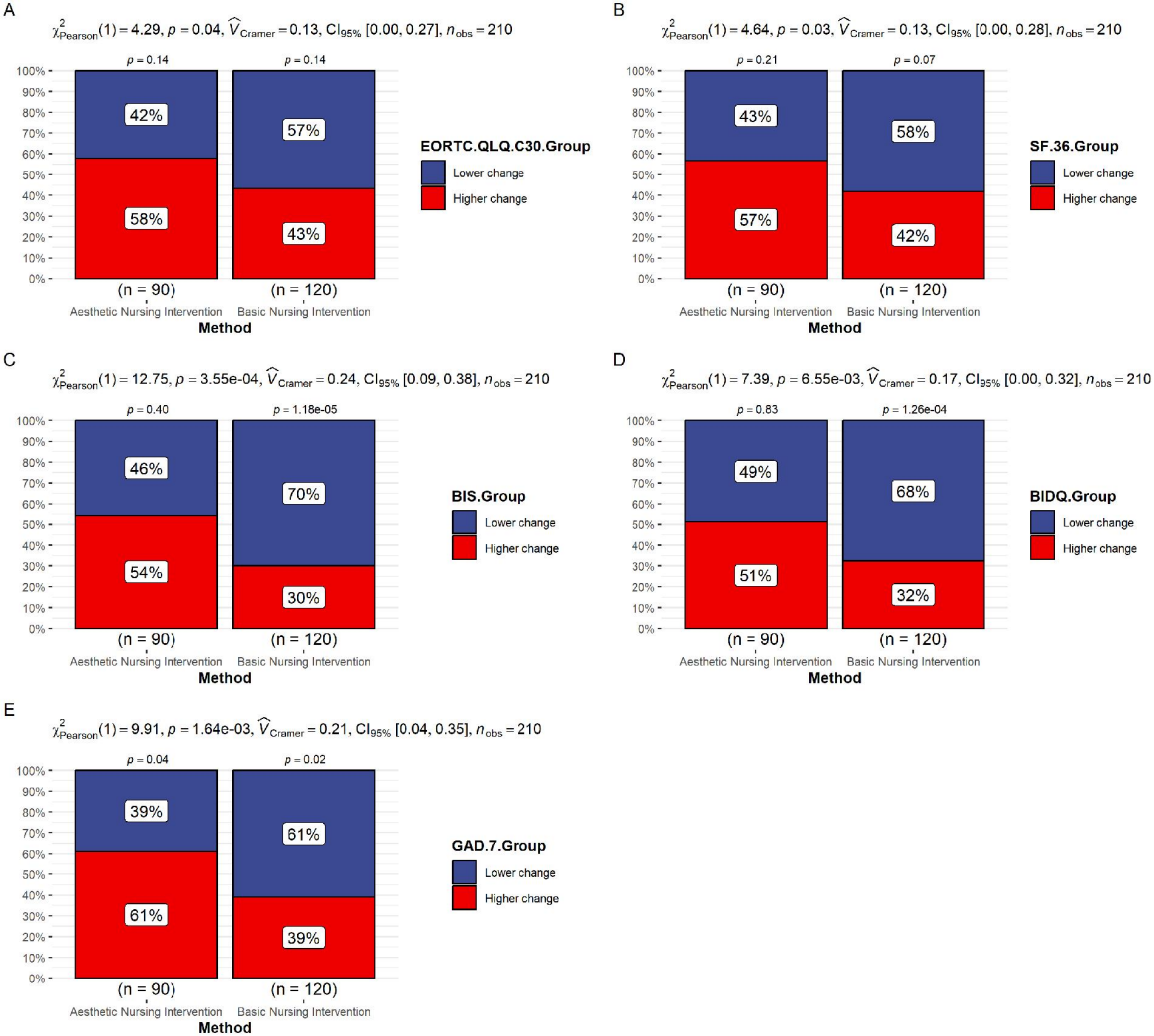
Differences in changes in **(A)** EORTC QLQ-C30, **(B)** SF-36, **(C)** body image scale, **(D)** body image disturbance questionnaire, and **(E)** GAD-7 scores between the aesthetic nursing intervention group and the basic nursing intervention group.

As an exploratory analysis, PHQ-9 was excluded because the intervention effect on depressive symptoms was not statistically significant in the regression model. Patients were then divided into high-change and low-change groups according to the median changes in EORTC QLQ-C30, SF-36, BIS, BIDQ, and GAD-7 scores across follow-up. The proportions of patients in these categories were compared between the aesthetic nursing group and the basic nursing group. The aesthetic nursing group showed a higher proportion of patients in the high-change category across these outcomes (Figures 1A–E), suggesting a broader pattern of postoperative improvement. However, these findings should be interpreted as exploratory.

### 3.6 Exploratory stratified analysis

**Figure 2:**
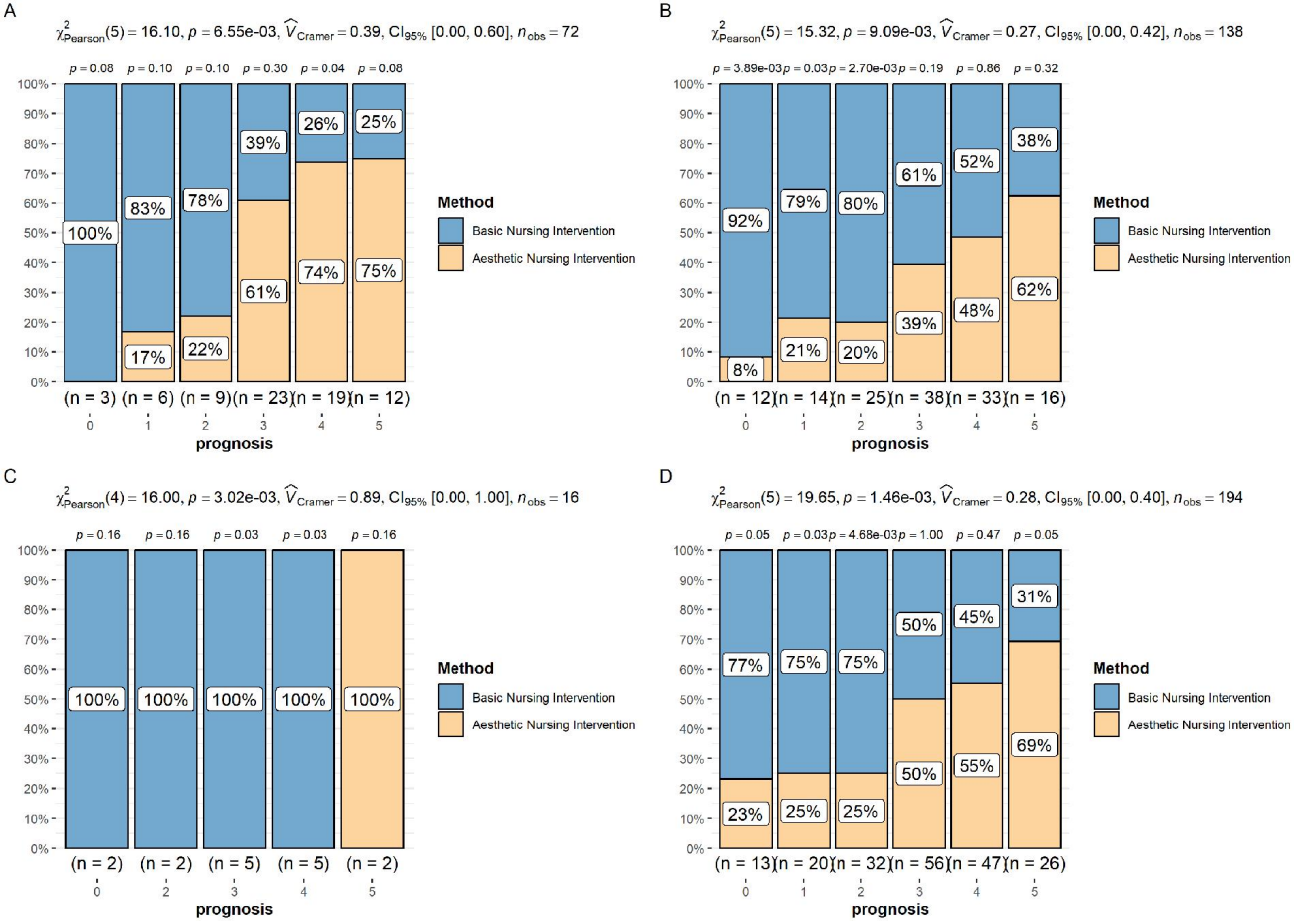
Proportions of patients in the aesthetic nursing group across different numbers of high-change indicators in the **(A)** open surgery subgroup, **(B)** laparoscopic surgery subgroup, **(C)** subgroup with intraoperative complications, and **(D)** subgroup without intraoperative complications.

Because significant baseline differences were observed in surgical approach and intraoperative complications, exploratory stratified analyses were further performed. Within each stratum, patients were classified according to the number of indicators belonging to the high-change group, as defined in Section 3.5. Across strata of surgical approach and intraoperative complications, the aesthetic nursing group consistently showed a higher proportion of patients with a greater number of high-change indicators (Figures 2A–D). These exploratory findings suggest that the overall pattern of benefit associated with aesthetic nursing remained broadly consistent across strata of surgical approach and intraoperative complications.

## 4 Discussion

This study explored the association between aesthetic nursing intervention and postoperative quality of life, body image, and psychological outcomes in patients with cervical cancer. The findings suggest that aesthetic nursing may be associated with better postoperative quality of life, more favorable body image outcomes, and reduced anxiety symptoms. These observations are broadly consistent with previous studies emphasizing the importance of body image, self-esteem, and supportive care in postoperative rehabilitation among women with gynecologic or breast cancer (16, 17).

At 2 weeks post-surgery, multiple outcomes reached their least favorable levels, suggesting that this may represent a clinically sensitive period for targeted supportive care. After this time point, most measures gradually improved, indicating a transition from acute postoperative burden to a recovery phase. The observed turning point may help inform the timing of supportive care and the planning of stage-appropriate interventions.

Firstly, improvement in quality-of-life outcomes appeared more evident in the aesthetic nursing group, especially after 2 weeks postoperatively, but the improvement effect before 2 weeks postoperatively was not significant. This indicates that aesthetic nursing cannot significantly improve patients' declining health-related quality of life in the short term after surgery, but can significantly improve patients' health-related quality of life in long-term follow-up. This may be because in the short term after cervical cancer surgery, patients’ bodies need to undergo a physiological recovery process. Postoperative pain, wound healing, and treatment-related adverse effects associated with chemotherapy or radiotherapy, such as fatigue, loss of appetite, and nausea, may directly impair postoperative quality of life (18, 19). These physiological discomforts and complications often have a significant negative impact on the health-related quality of life of patients in the short term. Aesthetic nursing helps patients gradually regain confidence and self-acceptance by paying attention to their appearance and emotional needs. However, this effect is often gradual and takes some time to show significant improvement. In the early postoperative period, patients may not have fully adapted to aesthetic nursing interventions, so they may not feel significant improvement in health-related quality of life in the short term. But as patients gradually adjust their psychology, recover physically, and adapt to nursing care, the nursing effect during long-term follow-up becomes more significant, and can continuously improve their health-related quality of life.

Secondly, improvements in body image were significantly more prominent in the aesthetic nursing group. This group demonstrated greater reductions in body image scores two weeks postoperatively and earlier improvements in body image disturbance scores. Enhancing body image is particularly important for cervical cancer patients, especially considering that surgical treatment often involves the removal of the uterus or other physiological changes. Such surgical changes can lead to negative self-perceptions and emotional distress. Patients may experience anxiety and discomfort related to their appearance and bodily functions, including feelings of inferiority or maladjustment (20, 21). Aesthetic nursing, as an innovative care model, focuses on both patients' appearance-related and emotional needs. It provides personalized services such as body image restoration, emotional support, and image adjustment guidance. These measures may enhance self-efficacy and strengthen patients' sense of control over appearance-related concerns, thereby reducing appearance-related anxiety. Moreover, emotional companionship from caregivers and peer support may help strengthen patients' social connections and sense of belonging, alleviating feelings of loneliness and shame. This holistic approach facilitates body–mind integration, enhances self-acceptance, and ultimately reduces body image distress. Through aesthetic nursing, patients are better able to adjust their body perception, restore a positive self-image, and psychologically emerge from the shadow of treatment, leading to improved postoperative well-being and health-related quality of life.

In terms of mental health, the relief of anxiety symptoms was more pronounced in the aesthetic nursing intervention group. This study found that before the turning point, patients' anxiety symptoms showed an upward trend, and aesthetic nursing could significantly alleviate early postoperative anxiety symptoms. This may be because aesthetic nursing helps patients feel understood and accepted by providing personalized care and emotional support. Such support may help relieve emotional tension and anxiety in the short term, especially in the early postoperative period, where patients often face physical discomfort, uncertainty about the future, and fear of cancer recurrence (22). The care, listening, and encouragement of nursing staff can provide psychological comfort to patients, thereby quickly relieving anxiety (23).

It is worth noting that the baseline PHQ-9 scores in this study were already at a relatively low level (median = 4, IQR: 3–6), and throughout the follow-up period, the highest score recorded was only 8. This indicates that the patients generally exhibited mild or no depressive symptoms prior to surgery. The core components of the aesthetic nursing intervention focused on image reconstruction, psychological counseling, and mindfulness meditation, but did not specifically target cognitive restructuring or enhancement of self-efficacy related to depression. Therefore, the improvement in depressive symptoms was not statistically significant. For patients with persistent or moderate-to-severe depressive symptoms, it may be necessary to incorporate additional psychological interventions, such as cognitive behavioral therapy, or to consider pharmacological support under psychiatric supervision.

Across follow-up, quality of life, body image, and anxiety outcomes all changed in a favorable direction, with generally greater improvement observed in the aesthetic nursing group than in the basic nursing group. The EORTC QLQ-C30 score increased by 22 points over time, and the supplementary SF-36 score increased by 24 points. Previous studies have suggested that a change of more than 10 points in EORTC QLQ-C30 may be clinically meaningful (24), and the magnitude of change observed in the present study therefore supports the possibility of clinically relevant improvement in postoperative quality of life.

For body image outcomes, both BIS and BIDQ scores decreased by 16 points over the follow-up period in the aesthetic nursing group, indicating a substantial reduction in body image burden, although no universally accepted minimally clinically important difference has been established for these measures. Similarly, GAD-7 scores decreased by 7 points in the aesthetic nursing group and by 5 points in the basic nursing group; given that a 3-point change has been reported as clinically meaningful (25), these findings suggest that anxiety symptoms improved to a potentially meaningful extent, particularly in the aesthetic nursing group. Taken together, these patterns support the interpretation that aesthetic nursing may contribute to clinically relevant improvement in several postoperative domains, although these observations should still be interpreted cautiously in light of the retrospective study design.

A strength of this study is that it identified two postoperative phases in the trajectories of quality of life, body image, and psychological symptoms, and explored the role of aesthetic nursing across these phases. Several limitations should be acknowledged. First, this was a single-center retrospective study, which may limit generalizability and introduce selection bias. Second, the groups were not randomized, and baseline differences in surgical approach and intraoperative complications were present. Although these variables were adjusted for in regression models and explored in stratified analyses, residual confounding cannot be excluded. Third, the follow-up duration was limited to 6 months and may not fully capture longer-term changes in body image and psychological well-being. Finally, some exploratory analyses should be interpreted cautiously and require confirmation in prospective studies.

## 5 Conclusion

In conclusion, aesthetic nursing may offer potential benefits in improving postoperative quality of life, body image, and anxiety in patients with cervical cancer. However, its association with depressive symptoms appeared limited in the present study, and the findings should be interpreted in light of the retrospective design and potential residual confounding. Further prospective studies with more rigorous designs are warranted.

## Data Availability

The raw data supporting the conclusions of this article will be made available by the authors, without undue reservation.
