# Supplementary material for: Effect of aesthetic nursing interventions on the health-related quality of life of cervical cancer patients after treatment
Source: Front Surg. 2026 May 29;13:1609977. doi: 10.3389/fsurg.2026.1609977 (PMC13260555; doi:10.3389/fsurg.2026.1609977)
Supplement: Supplementary file 1 [file Table1.docx]

**Table S1.** Comparison of longitudinal changes in quality of life between two groups of patients using a linear mixed-effects model.

| **EORTC QLQ-C30** | **β** | **SE** | **95% CI** | **p value** |
| --- | --- | --- | --- | --- |
| **Group Aesthetic** | -0.401 | 0.377 | −1.22–0.41 | 0.334 |
| **Time 2w (2 weeks post-surgery)** | -6.081 | 0.415 | −6.82–−5.34 | < 0.001 |
| **Time 1m (1 month post-surgery)** | -0.851 | 0.377 | −1.59–−0.11 | 0.024 |
| **Time 3m (3 month post-surgery)** | 8.191 | 0.377 | 7.45–8.93 | < 0.001 |
| **Time 6m (6 month post-surgery)** | 22.665 | 0.377 | 21.93–23.41 | < 0.001 |
| **Group Aesthetic** × **Time 2w** | 1.053 | 0.576 | −0.08–2.18 | 0.068 |
| **Group Aesthetic** × **Time 1m** | 1.110 | 0.576 | −0.02–2.24 | 0.054 |
| **Group Aesthetic** × **Time 3m** | 1.529 | 0.576 | 0.40–2.66 | 0.008 |
| **Group Aesthetic** × **Time 6m** | 1.674 | 0.576 | 0.54–2.80 | 0.004 |
|  |  |  |  |  |
| **SF-36** | **β** | **SE** | **95% CI** | **p value** |
| **Group Aesthetic** | -0.612 | 0.365 | −1.33–0.11 | 0.094 |
| **Time 2w (2 weeks post-surgery)** | -4.507 | 0.337 | −5.17–−3.85 | < 0.001 |
| **Time 1m (1 month post-surgery)** | 4.123 | 0.337 | 3.46–4.79 | < 0.001 |
| **Time 3m (3 month post-surgery)** | 13.218 | 0.337 | 12.56–13.88 | < 0.001 |
| **Time 6m (6 month post-surgery)** | 23.988 | 0.337 | 23.33–24.65 | < 0.001 |
| **Group Aesthetic** × **Time 2w** | 1.848 | 0.515 | 0.84–2.86 | < 0.001 |
| **Group Aesthetic** × **Time 1m** | 1.657 | 0.515 | 0.65–2.67 | 0.001 |
| **Group Aesthetic** × **Time 3m** | 2.364 | 0.515 | 1.35–3.38 | < 0.001 |
| **Group Aesthetic** × **Time 6m** | 1.735 | 0.515 | 0.72–2.75 | 0.001 |
