# Supplementary figures and images for: Effect of aesthetic nursing interventions on the health-related quality of life of cervical cancer patients after treatment
Source: Front Surg. 2026 May 29;13:1609977. doi: 10.3389/fsurg.2026.1609977 (PMC13260555; doi:10.3389/fsurg.2026.1609977)

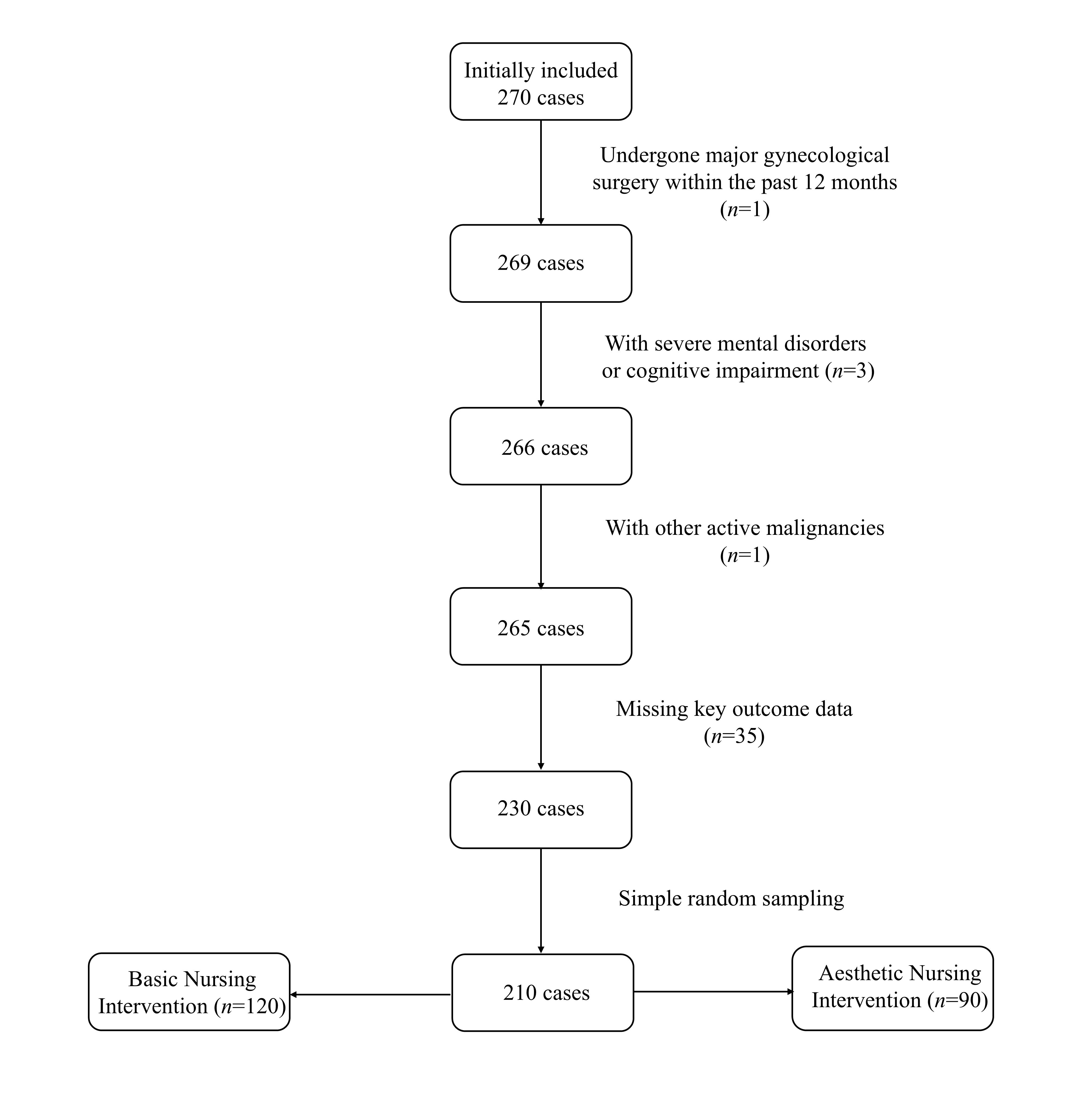

Supplement: Supplementary file 2 [file Image1.tif]
